# Conditioning-induced expression of novel glucose transporters in canine skeletal muscle homogenate

**DOI:** 10.1371/journal.pone.0285424

**Published:** 2023-05-03

**Authors:** Montana Renae Barrett, Michael Scott Davis

**Affiliations:** Department of Physiological Sciences, College of Veterinary Medicine, Oklahoma State University, Stillwater, OK, United States of America; University of Minnesota Medical School, UNITED STATES

## Abstract

Athletic conditioning can increase the capacity for insulin-stimulated skeletal muscle glucose uptake through increased sarcolemmal expression of GLUT4 and potentially additional novel glucose transporters. We used a canine model that has previously demonstrated conditioning-induced increases in basal, insulin- and contraction-stimulated glucose uptake to identify whether expression of glucose transporters other than GLUT4 was upregulated by athletic conditioning. Skeletal muscle biopsies were obtained from 12 adult Alaskan Husky racing sled dogs before and after a full season of conditioning and racing, and homogenates from those biopsies were assayed for expression of GLUT1, GLUT3, GLUT4, GLUT6, GLUT8, and GLUT12 using western blots. Athletic conditioning resulted in a 1.31 ± 0.70 fold increase in GLUT1 (p <0.0001), 1.80 ± 1.99 fold increase in GLUT4 (p = 0.005), and 2.46 ± 2.39 fold increase in GLUT12 (p = 0.002). The increased expression of GLUT1 helps explain the previous findings of conditioning-induced increases in basal glucose clearance in this model, and the increase in GLUT12 provides an alternative mechanism for insulin- and contraction-mediated glucose uptake and likely contributes to the substantial conditioning-induced increases in insulin sensitivity in highly trained athletic dogs. Furthermore, these results suggest that athletic dogs can serve as a valuable resource for the study of alternative glucose transport mechanisms in higher mammals.

## Introduction

Glucose is a vital fuel source for all cellular processes. For glucose to be metabolized to a useable fuel it must first be transported into the cell. Cellular uptake of glucose occurs through facilitated diffusion mediated by various glucose transporters, with up to 14 isoforms having been reported in different tissues and species [1]. In most cells, basal glucose transport occurs through transporters such as GLUT1 that are expressed continually on the cell surface. In addition, glucose uptake in insulin-responsive tissues utilized glucose transporter isoforms such as GLUT4 that are stored in intracellular vesicles that fuse with the cell membrane to permit facilitated diffusion of glucose. Fusion of intracellular vesicles containing GLUT4 with the sarcolemma in skeletal muscle also can be stimulated by muscle contraction to provide acute increases in glucose availability to fuel exercise [2]. Regular exercise increases insulin-mediated glucose clearance [3–5]. Skeletal muscle GLUT4 content is increased by athletic conditioning [6, 7], and emerging evidence suggests that other glucose transporters may also play a role in skeletal muscle glucose transport in exercise-trained subjects [8].

Racing Alaskan sled dogs are an extreme example of highly conditioned ultra-endurance athletes, and glucose homeostasis has been studied extensively in this population with surprising results. Athletic conditioning of sled dogs results in increases in both insulin sensitivity and insulin independent peripheral glucose uptake [9, 10], suggesting that conditioning can increase expression of both translocatable and basal glucose transporters. Athletic conditioning of racing sled dogs also increased both basal and exercise-induced glucose transport in skeletal muscle giant sarcolemmal vesicles [11]. However, that study found a DECREASE in the expression of GLUT4 in those vesicles compared to vesicles from rested dogs, suggesting that the increase in exercise-induced glucose uptake was due to a different glucose transporter that also translocates in response to exercise. Based on these findings, our hypothesis was that in addition to the well-documented glucose transporter GLUT 4, there are several other glucose transporters involved in the uptake of glucose in the skeletal muscle of highly-conditioned athletes. Therefore, we conducted a study in a population of racing Alaskan sled dogs to determine the conditioning induced changes in skeletal muscle expression of glucose transporters.

## Materials/Methods

**Fig 1:**
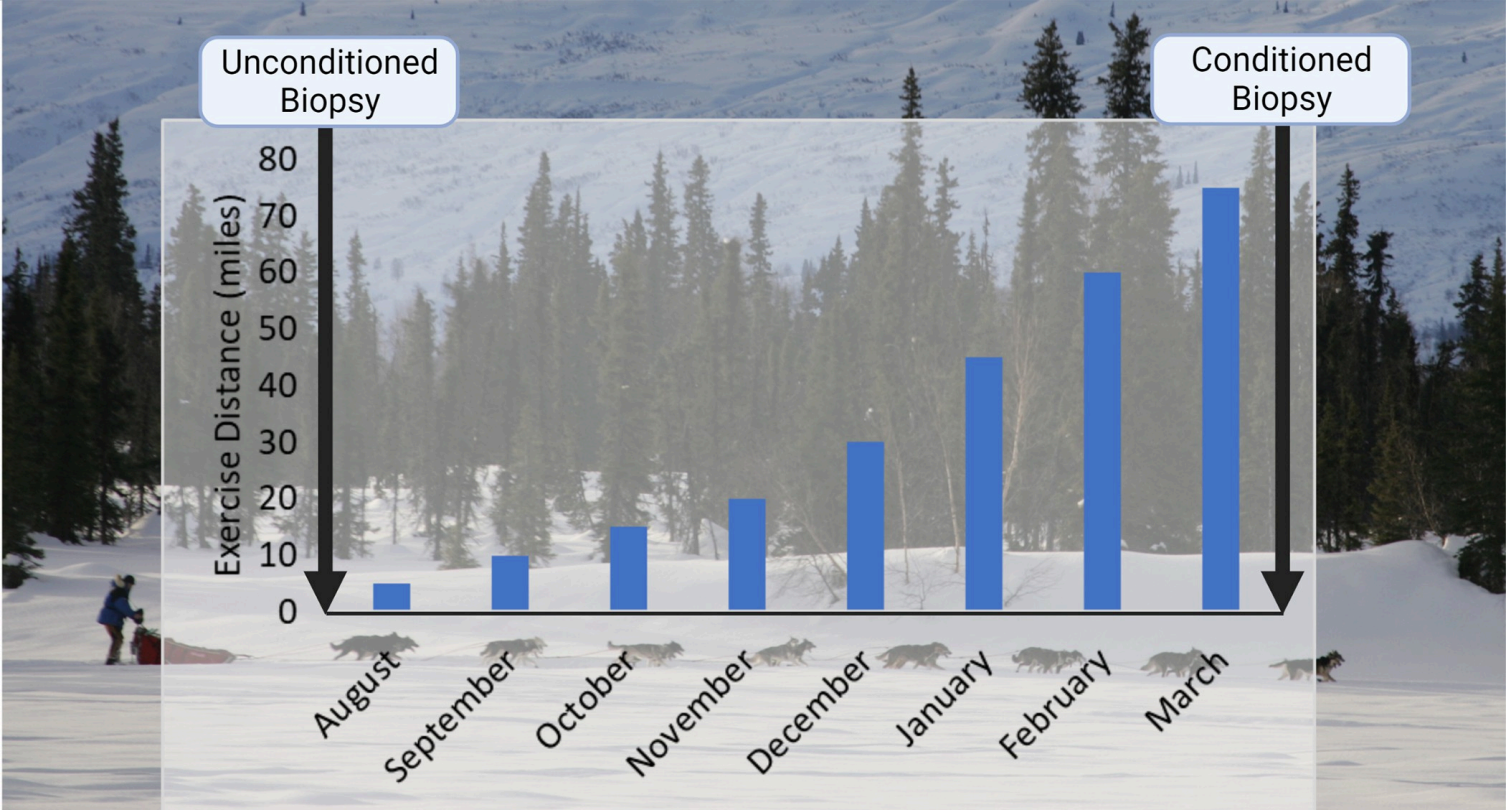
Schematic of study design. All dogs were biopsied prior to initiating progressive overload training for endurance exercise. Average duration of daily workouts increased from approximately 5 miles/workout (workouts every 2–3 days) at the beginning of conditioning to 75 miles/workout (every 3–4 days) at the conclusion of conditioning. All dogs were rested for at least 48 hr prior to the Conditioned biopsy.

All proposed study designs and methods were approved by the Oklahoma State University Institutional Animal Care and Use Committee. Twelve Alaskan sled dogs from a single kennel (median age 4.5 years (range 2–7 yrs), 3 females, 3 neutered males, and 6 intact males, body mass 24.6±3.2 kg) were used for this study. Skeletal muscle biopsies were obtained at two time points: Conditioned, when dogs had undergone 7 mo of progressive endurance conditioning and Unconditioned, when dogs were at minimal fitness having had no compulsory exercise for at least 4 mo (Fig 1). Conditioning consisted of exercise sessions in groups of 12–16 dogs connected to a single gangline and either a small all-terrain vehicle or sled (depending on trail conditions) with speed limited to 16–18 km/h to manage the production and dissipation of metabolic heat. Conditioning intensity results from increasing workout distance, and the decision to increase distance is made via monitoring the voluntary speed of the team via GPS: if dogs maintained within 10% of the maximum allowed speed during the last 30 min of the workout, then the distance of the next workout is increased by 25%. To obtain the muscle biopsies, the dogs were placed under general anesthesia using propofol (PropoFlo™ 200mg/20mL, ZOETIS) administered as an intravenous bolus (approximately 7mg/kg, adjusted to the desired effect). Biopsies were taken from the biceps femoris using a percutaneous biopsy needle (14-gauge E-Z Core single action biopsy needle, Products Group International, Lyons, CO) to yield ~80 mg of wet muscle, a portion of which was immediately snap frozen for western blot analysis. Frozen muscle was homogenized on ice in ~2 ml lysis buffer (20 mM HEPES (pH7.4), 100 mM potassium chloride, 2 mM ethylene-diamine-tetraacetic acid, 10 mM sodium fluoride, 10 mM sodium pyrophosphate, 2 mM sodium orthovanadate, 1 mM ammonium molybdate, 1 mM phenylmethylsulfonyl fluoride) per 0.2 g of tissue using a Dounce homogenizer and tissue shredder. Protein concentration was measured using Coomassie blue protein assay, and samples to be analyzed were diluted to a concentration of 2 ng/μl using lysis buffer and 2x Laemmli buffer (65.8 mM TRIS-HCl (pH6.8), 2.1% SDS, 26.3% glycerol, 0.01% bromophenol blue). Samples were run in duplicate on a 10% SDS-polyacrylamide gel at 200 V and 0.025 mA/gel for ~1.25 h at 4°C, with both Unconditioned and Conditioned samples from an individual subject on the same gel. Total mass of protein loaded on the gel, and the specific primary antibody and antibody dilution for each target is found in Table 1. Primary antibodies were selected based on manufacturer claims of cross-reactivity and presence of target epitope (when known) in BLAST-predicted canine amino acid sequence. Further validation was based on the presence of primary detected band at approximate molecular weight of predicted canine target and a linear relationship between gel protein load and target band immunodensity. The latter step was used to select the total protein load for western blot analysis of transporter abundance in the study samples. The gel was transferred overnight onto an Immobilon-FL PVDF membrane in transfer buffer at 30 V and 0.09 mA for 16 h. Membranes were blocked for 1 h using Rockland Blocking Buffer (MB-070), incubated in the target primary antibody for 1 h, and incubated in a secondary antibody (LI-COR IRDye 680RD anti-rabbit 926–68073 (1:10,000); LI-COR 680RD anti-goat 926–68074 (1:10,000)) for 1 h, all at room temperature. Between each incubation the membrane was washed four times with 0.1% TBS-T for 5 min each wash. The membrane was washed a final time for 5 min in TBS and immediately scanned using a LI-COR Odyssey imager. Relative target band intensity was measured using commercial software (LI-COR Empiria Studio 1.3) and expressed as average intensity corrected for the average intensity of β-actin in the sample. The effect of conditioning was determined using a 2-way repeated measures ANOVA, with fitness (unconditioned, conditioned) and sex (female, male-neutered, male-intact) as independent variables and subject as a blocking variable. A p<0.05 was considered statistically significant.

**Table 1:** Western blot methods for detection of glucose transporters in canine skeletal muscle homogenate.

Target	Gel Load	Primary Antibody Vendor, Catalog Number, and Antibody Registration Number (antibodyregistry.org)	Primary Antibody Dilution
GLUT1	20 μg	Abcam ab115730 RRID: AB_10903230	1:1000
GLUT3	N/A	Aviva Systems Biology ARP63350_P050	1:1000
GLUT4	20 μg	Abcam ab33780 RRID: AB_2191441	1:1000
GLUT6	30 μg	Aviva Systems Biology ARP43973_P050 RRID: AB_2046944	1:1000
GLUT8	10 μg	Aviva Systems Biology OAEB00492 RRID: AB_10877183	1:1000
GLUT12	20 μg	Atlas Antibodies HPA031593 RRID: AB_10600810	1:200
β-actin	30 μg	LI-COR 926–42210 RRID: AB_1850027	1:1000

## Results

**Fig 2:**
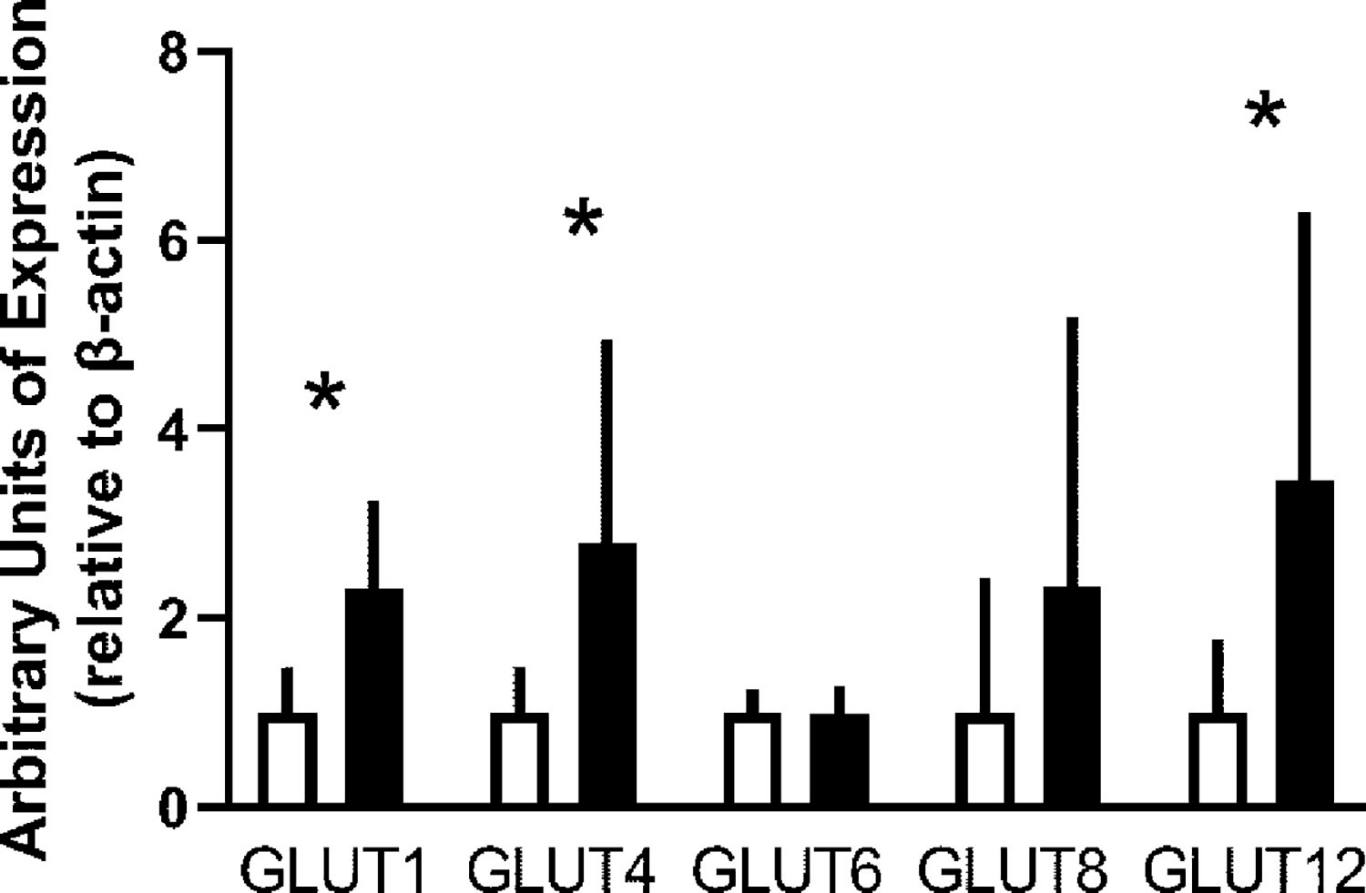
Effect of athletic conditioning on expression of glucose transporters in canine skeletal muscle. N = 12 subjects with biopsies obtained prior to and after 7 months of progressive aerobic conditioning. Light bars: Unconditioned. Dark bars: Conditioned. For purposes of visual clarity, expression data for individual dogs were divided by the mean of all unconditioned dogs, then plotted as mean + standard deviation. * effect of conditioning, p < 0.05.

**Fig 3:**
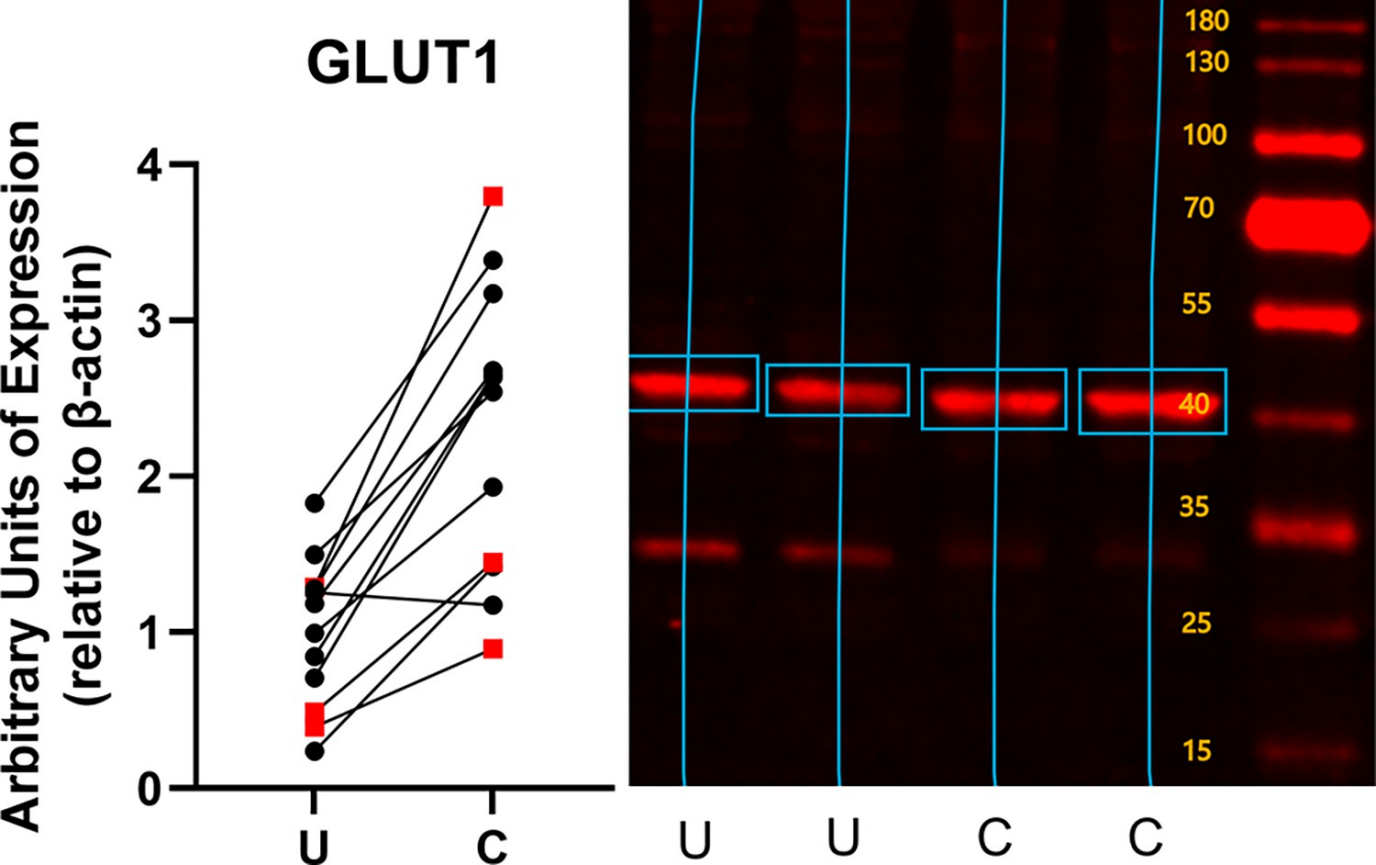
Effect of athletic conditioning on expression of GLUT1 in canine skeletal muscle, with representative western blot image showing primary band used for quantification of expression. N = 12 subjects with biopsies obtained prior to and after 7 months of progressive aerobic conditioning. Male subjects (n = 9) displayed as black circles and female subjects (n = 3) displayed as red squares. U: Unconditioned; C: Conditioned. Effect of conditioning, p = 0.0004.

**Fig 4:**
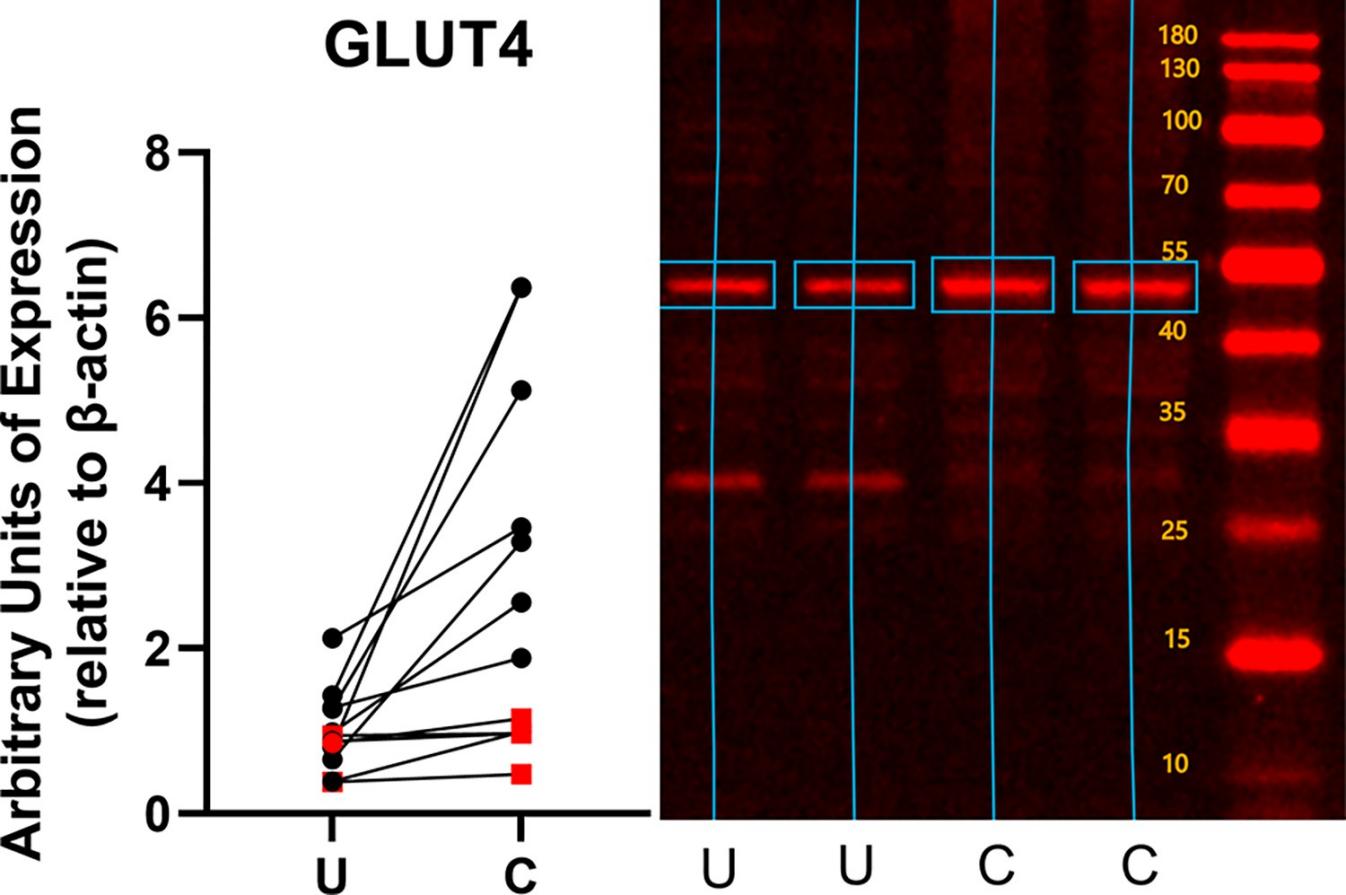
Effect of athletic conditioning on expression of GLUT4 in canine skeletal muscle, with representative western blot image showing primary band used for quantification of expression. N = 12 subjects with biopsies obtained prior to and after 7 months of progressive aerobic conditioning. Male subjects (n = 9) displayed as black circles and female subjects (n = 3) displayed as red squares. U: Unconditioned; C: Conditioned. Effect of conditioning, p = 0.023.

**Fig 5:**
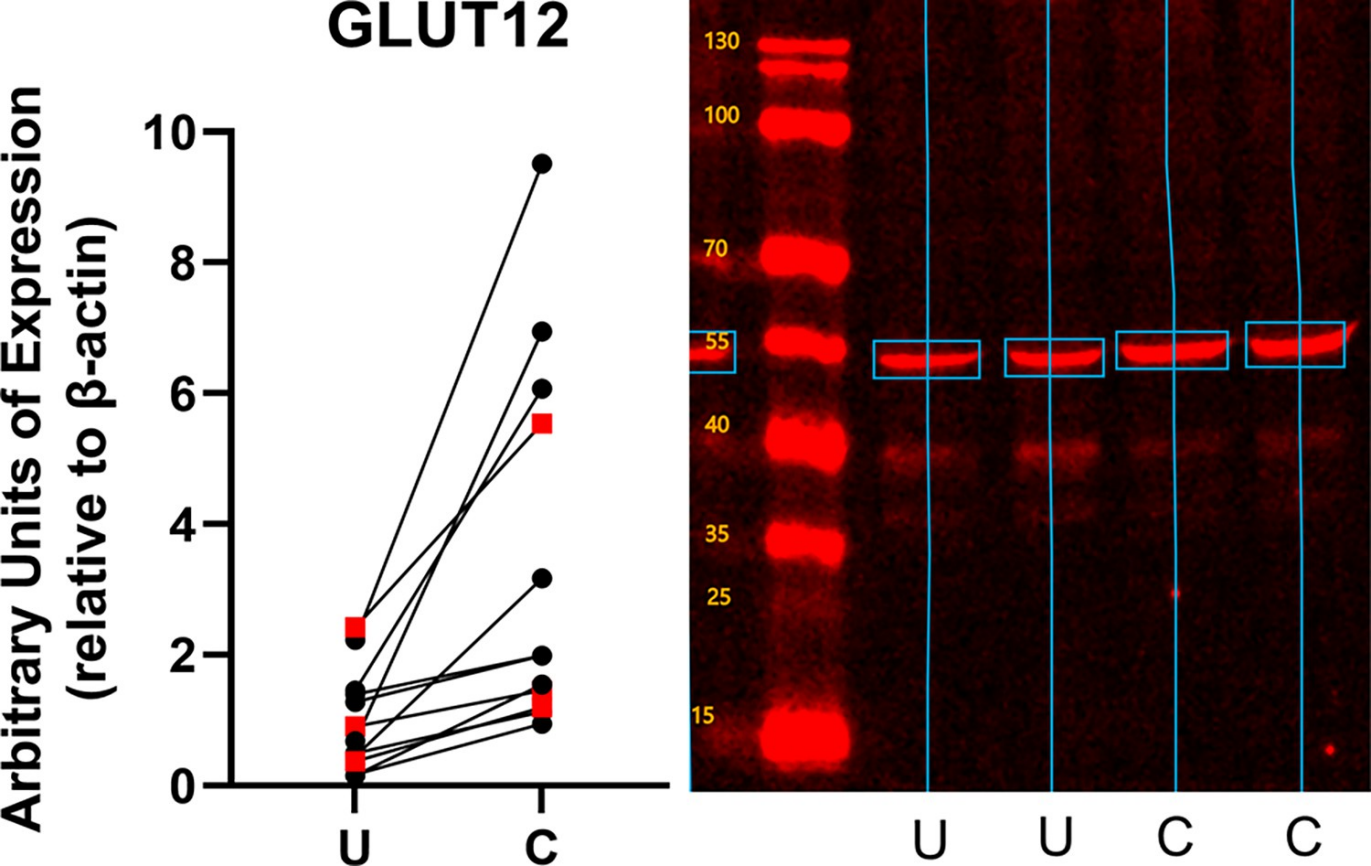
Effect of athletic conditioning on expression of GLUT12 in canine skeletal muscle, with representative western blot image showing primary band used for quantification of expression. N = 12 subjects with biopsies obtained prior to and after 7 months of progressive aerobic conditioning. Male subjects (n = 9) displayed as black circles and female subjects (n = 3) displayed as red squares. U: Unconditioned; C: Conditioned. Effect of conditioning, p = 0.017.

**Fig 6:**
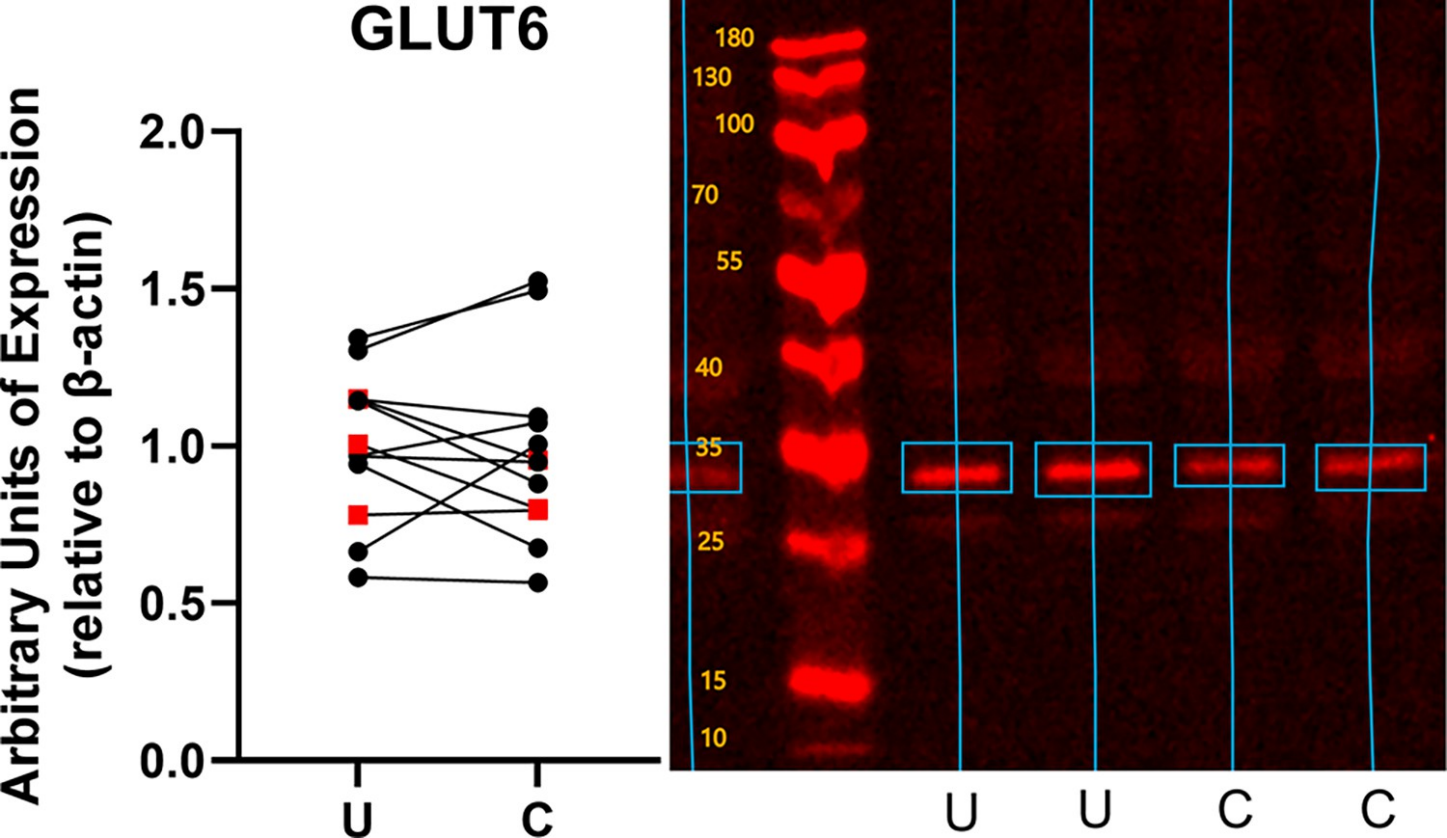
Effect of athletic conditioning on expression of GLUT6 in canine skeletal muscle, with representative western blot image showing primary band used for quantification of expression. N = 12 subjects with biopsies obtained prior to and after 7 months of progressive aerobic conditioning. Male subjects (n = 9) displayed as black circles and female subjects (n = 3) displayed as red squares. U: Unconditioned; C: Conditioned. Effect of conditioning, p = 0.79.

**Fig 7:**
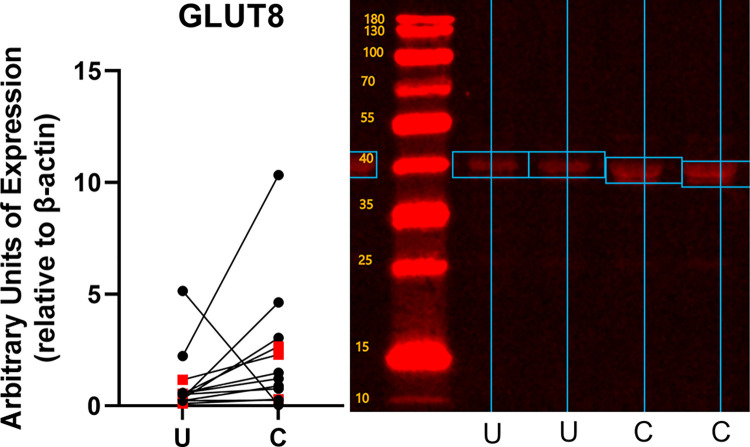
Effect of athletic conditioning on expression of GLUT8 in canine skeletal muscle, with representative western blot image showing primary band used for quantification of expression. N = 12 subjects with biopsies obtained prior to and after 7 months of progressive aerobic conditioning. Male subjects (n = 9) displayed as black circles and female subjects (n = 3) displayed as red squares. U: Unconditioned; C: Conditioned. Effect of conditioning, p = 0.17.

Conditioning increased the expression of both constitutive and inducible glucose transporters (Fig 2). GLUT1 expression increased by 1.31 ± 0.70 fold (mean ± standard deviation, p = 0.0004) over the level of expression in unconditioned subjects (Fig 3). GLUT 4 expression increased by 1.80 ± 1.99 fold (p = 0.023) (Fig 4), and GLUT12 expression increased 2.46 ± 2.39 fold (p = 0.017) over the level of expression in unconditioned subjects (Fig 5). We did not detect a statistically significant increase in GLUT 6 (-0.015 ± 0.20 fold increase compared to unconditioned, p = 0.79) (Fig 6) or GLUT 8 (1.33 ± 3.07 fold increase compared to unconditioned, p = 0.17) (Fig 7) as an effect of conditioning, and were unable to detect expression of GLUT3 in our canine skeletal muscle samples. There was no effect of sex on expression of any glucose transporter.

## Discussion

The significant finding of this study was the induction of several novel glucose transporters by prolonged aerobic conditioning in addition to the expected conditioning-induced increase in expression of GLUT4. While widely accepted to be the primary glucose transporter within skeletal muscle [2], a previous study in GLUT4 knockout mice demonstrated that there was no impairment of glucose uptake with the elimination of this glucose transporter [12]. That study found that GLUT1, GLUT3, GLUT6, and GLUT10 protein levels increased in response to the combination of knocking out GLUT4 and biomechanical overload of selected skeletal muscles, suggesting an involvement of one or more of these glucose transporters in the preservation of skeletal muscle glucose uptake.

It is well documented that athletic conditioning increases GLUT4 content of skeletal muscle. Expression of skeletal muscle GLUT4 content was increased 1.4–1.7 fold by 6 weeks of treadmill exercise in rats, with the increased expression limited to oxidative (red) muscles [13]. A similar increase in rat skeletal muscle GLUT4 expression was reported by Goodyear et al [14], who also demonstrated an increase in insulin-mediated sarcolemmal expression and glucose transport in trained rats. Comparable results have been reported in humans, with increased skeletal muscle GLUT4 expression [3, 8, 15, 16] and insulin-mediated glucose transport in skeletal muscle [17] due to athletic training. Although a previous study in athletically conditioned dogs failed to demonstrate an increase in skeletal muscle GLUT4 expression [11], the current study is consistent with the studies in other species both qualitatively and quantitatively. Despite the increase in insulin-mediated sarcolemmal glucose transport, endurance conditioning results in decreased exercise-induced glucose transport in humans [18], which stands in contrast to previous work in endurance-trained dogs showing an increase in contraction-mediated glucose transport. However, that study of athletic dogs failed to demonstrate an increase in skeletal muscle GLUT4 expression and reported a DECREASE in contraction mediated sarcolemmal expression of GLUT4, suggesting that in trained dogs, the increase in contraction-mediated sarcolemmal glucose transport (and perhaps also insulin-mediated sarcolemmal glucose transport) is due to a glucose transporter other than GLUT4.

The results of this study support the conclusion that GLUT12 is that unidentified glucose transporter. GLUT12 is highly expressed in rat fetal tissues and appears to be responsible for glucose transport–particularly insulin-mediated glucose uptake–than GLUT4 which is expressed much later in fetal development [19]. GLUT12 has similar kinetic affinities for glucose as the constitutive transporter GLUT1, further suggesting the importance of the role GLUT12 has in maintaining glucose homeostasis within skeletal muscle [20]. Although GLUT4 is considered the primary insulin- and contraction-mediated glucose transporter in adult skeletal muscle, GLUT12 is increased in the skeletal muscle of trained cyclists and translocates to the sarcolemma in a manner similar to that of GLUT4 [8]. Additionally, there is evidence that at least in some tissues, GLUT12 can be constitutively expressed on the cell membrane to contribute to glucose uptake [21]. Thus, it is plausible to suggest that GLUT12 could play as important of a role in skeletal muscle glucose uptake as the more widely studied transporter GLUT4. The shared expression of GLUT4 and GLUT12 in human skeletal muscle, including very similar patterns of translocation to the sarcolemma in response to insulin stimulation, suggest that GLUT4 and GLUT12 may be co-localized in the same sarcoplasmic vesicles [8]. However, the results of the contraction-mediated expression studies in dogs raise the possibility of the different transporters localized in distinct vesicle populations with the GLUT4-containing vesicles being less sensitive to contraction-mediated translocation [11]. Further study is needed to evaluate the behavior of these transporters.

Our results showed a 1.31 ± 0.70 fold increase in GLUT1 due to aerobic conditioning. These findings provide some explanation in the conditioning-induced increased in insulin and contraction independent glucose transport demonstrated in the previously discussed studies [9, 11]. GLUT1 is a high affinity glucose transporter present at baseline and is responsible for ongoing glucose homeostasis, although GLUT4 may also play a role in unstimulated glucose transport in some canine disease models [22]. Studies of trained humans have failed to find a change in GLUT1 expression [8], but GLUT1 expression was increased in GLUT4 knockout mice and may have played a role in maintaining glucose uptake in that model [12]. Although increased expression of GLUT1 does not necessarily result in a proportional increase in basal glucose transport [23], the similarity between the magnitude of increased GLUT1 expression in this study and the magnitude of conditioning-induced increase in both whole-body [9] and muscle-specific [11] basal glucose transport is compelling.

The role of other facultative glucose transporters that have been associated with skeletal muscle is less certain. Although more commonly associated with liver and adipose tissue [24], GLUT8 expression is increased in equine skeletal muscle in response to 5-Aminoimidazole-4-carboxamide ribonucleotide (AICAR) administration [25] and may also be upregulated in humans following endurance exercise conditioning [26]. In this study, the effect of conditioning on skeletal muscle GLUT8 expression failed to achieve statistical significance, but this is likely at least partially an effect of limited statistical significance and will continue to be investigated in this model. Two other glucose transporters that have been associated with skeletal muscle were either not sufficiently expressed for detection by western blot (GLUT3) or expression was not significantly altered by conditioning (GLUT6). GLUT10 is a glucose transporter associated with mitochondrial membranes and is upregulated in GLUT4-knockout mice. Unfortunately, we were unable to locate suitable antibodies for detection of canine GLUT10, so we could not determine whether its expression is altered by endurance conditioning in these dogs.

The results of this study may help explain the metabolic strategies that produce the impressive exercise capacity of highly conditioned athletic dogs. The typical conditioning program for ultraendurance sled dog racing has been shown to increase muscle oxidative capacity [27, 28] without evidence of fiber type switching (based on myosin ATPase staining) [29]. Despite a diet that is strikingly high in fat [29, 30], conditioning for endurance exercise in these athletes results in a distinct glucocentric metabolic strategy [31] characterized by high stimulus for hepatic glucose output during exercise [32] and increased capacity for peripheral glucose clearance [9]. Increased expression of glucose transporters provide a critical step in support of skeletal muscle utilization of glucose as the preferred substrate for exercise [28, 33]. Demonstration of sarcolemmal expression of these transporters, either through immunohistochemistry or isolation of sarcolemmal membranes, is necessary to definitely show a role in the contraction- and insulin-induced changes in glucose metabolism in this model.

## Supporting information

S1 Raw images (PDF)

S1 Data (PDF)
