# Supplementary material for: Conditioning-induced expression of novel glucose transporters in canine skeletal muscle homogenate
Source: PLoS One. 2023 May 3;18(5):e0285424. doi: 10.1371/journal.pone.0285424 (PMC10155965; doi:10.1371/journal.pone.0285424)
Supplement: S1 Raw images — (PDF) [file pone.0285424.s001.pdf]

Supplemental Files for PONE-D-22-35267
Conditioning-induced induction of novel glucose transporters in canine skeletal muscle

Each sample was run in duplicate, with both Unconditioned (U) and Conditioned (C) for a given subject run on the same gel. Subject name and antibody target listed on each blot image. "Control" was a homogenized collection of samples used to initially validate and determine linear range of protein detection, and was included as a technical positive control but not used for any data processing or correction.
Images were acquired using a LiCor gel reader, using a 700 nm filter to capture fluorescent signal from the LiCor secondary antibodies. Raw images were imported into LiCor Empiria software for analysis, and exported as 600 dpi TIFF files using that software after detection and labelling of lanes, molecular weight markers, and target bands.

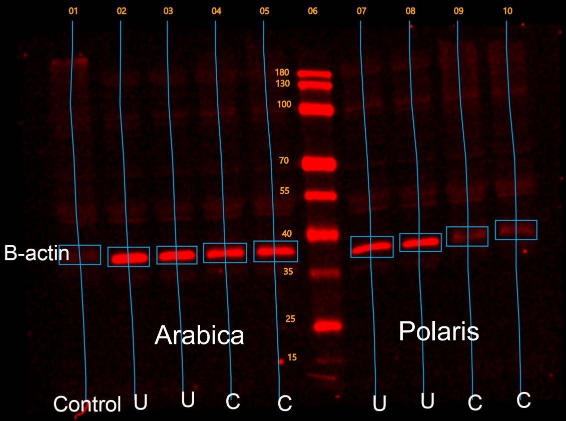
01
02
03
04
05
06
07
08
09
10
180
130
100
70
55
40
35
25
15
B-actin
Arabica
Polaris
Control
U
U
C
C
U
U
C
C

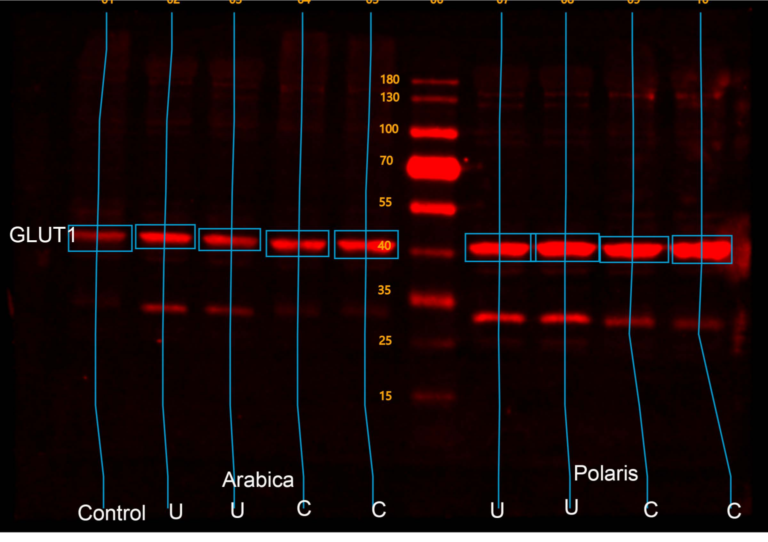

180
130
100
70
55
GLUT1
40
35
25
15
Arabica
Polaris
Control
U
U
C
C
U
U
C
C

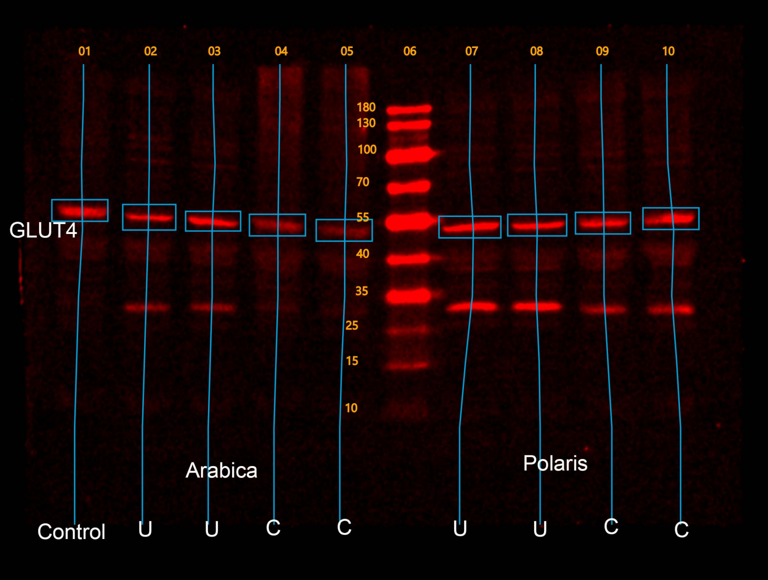
01
02
03
04
05
06
07
08
09
10
GLUT4
180
130
100
70
55
40
35
25
15
10
Arabica
Polaris
Control
U
U
C
C
U
U
C
C

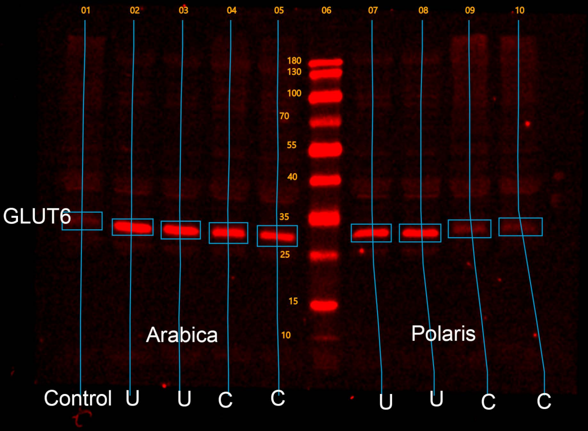
01
02
03
04
05
06
07
08
09
10
180
130
100
70
55
40
35
25
15
10
GLUT6
Arabica
Polaris
Control
U
U
C
C
U
U
C
C

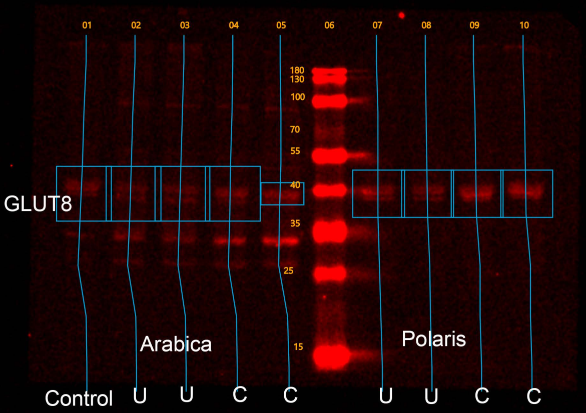
01
02
03
04
05
06
07
08
09
10
180
130
100
70
55
40
35
25
15
GLUT8
Arabica
Polaris
Control
U
U
C
C
U
U
C
C

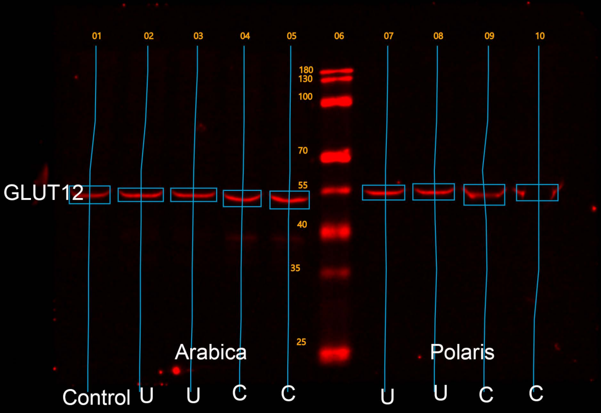
01
02
03
04
05
06
07
08
09
10
180
130
100
70
55
40
35
25
GLUT12
Arabica
Polaris
Control
U
U
C
C
U
U
C
C

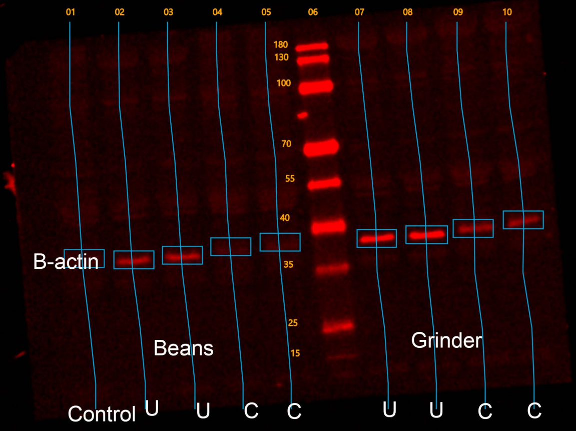
01
02
03
04
05
06
07
08
09
10
180
130
100
70
55
40
35
25
15
B-actin
Beans
Grinder
Control
U
U
C
C
U
U
C
C

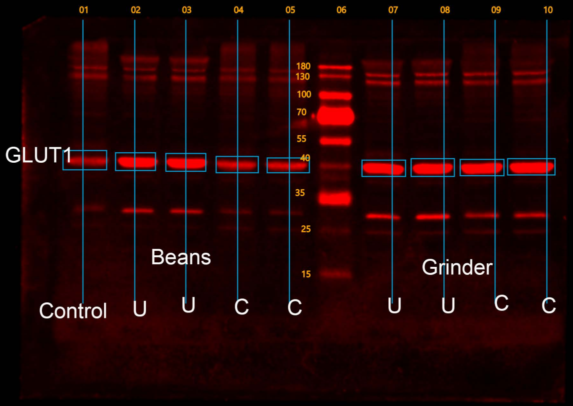
01
02
03
04
05
06
07
08
09
10
180
130
100
70
55
40
35
25
15
GLUT1
Beans
Grinder
Control
U
U
C
C
U
U
C
C

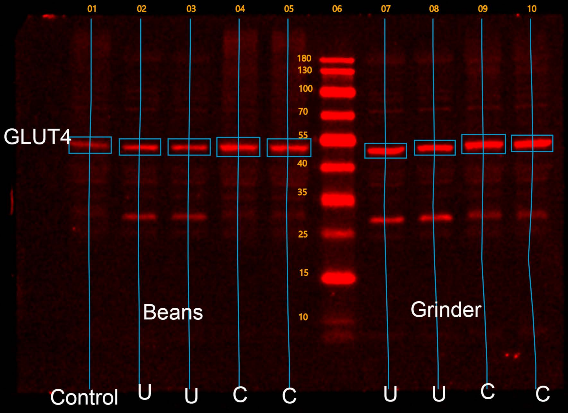
01
02
03
04
05
06
07
08
09
10
GLUT4
180
130
100
70
55
40
35
25
15
10
Beans
Grinder
Control
U
U
C
C
U
U
C
C

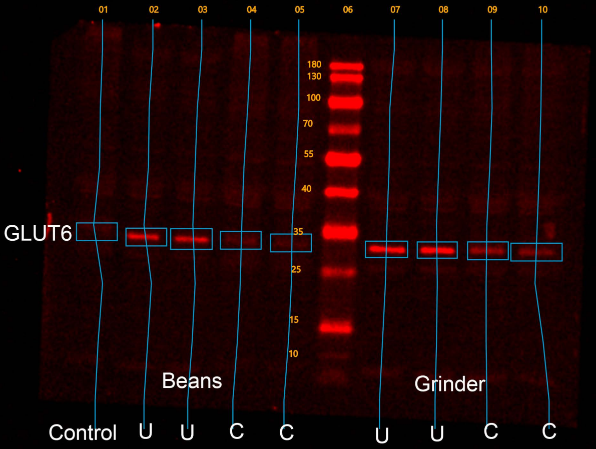
01
02
03
04
05
06
07
08
09
10
180
130
100
70
55
40
35
25
15
10
GLUT6
Beans
Grinder
Control
U
U
C
C
U
U
C
C

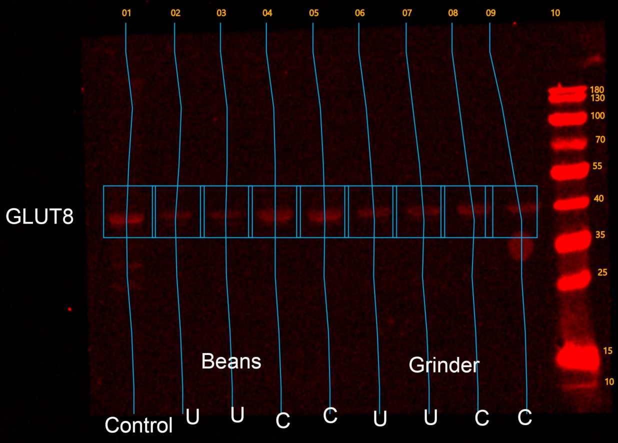
01
02
03
04
05
06
07
08
09
10
180
130
100
70
55
40
35
25
15
10
GLUT8
Beans
Grinder
Control
U
U
C
C
U
U
C
C

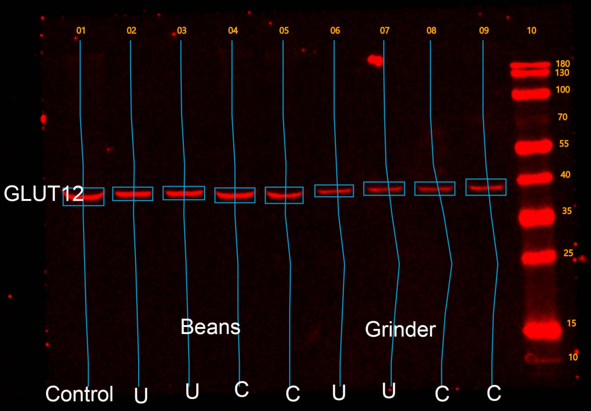
01
02
03
04
05
06
07
08
09
10
180
130
100
70
55
40
35
25
15
10
GLUT12
Beans
Grinder
Control
U
U
C
C
U
U
C
C

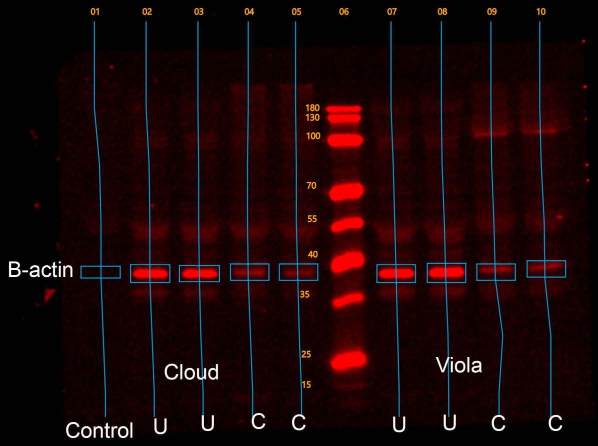

01
02
03
04
05
06
07
08
09
10
180
130
100
70
55
40
35
25
15
B-actin
Cloud
Viola
Control
U
U
C
C
U
U
C
C

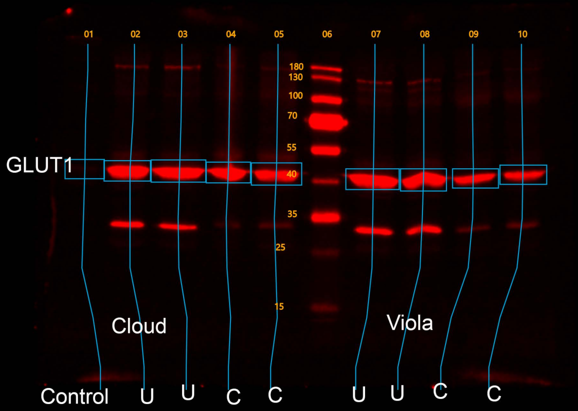
01
02
03
04
05
06
07
08
09
10
180
130
100
70
55
40
35
25
15
GLUT1
Cloud
Viola
Control
U
U
C
C
U
U
C
C

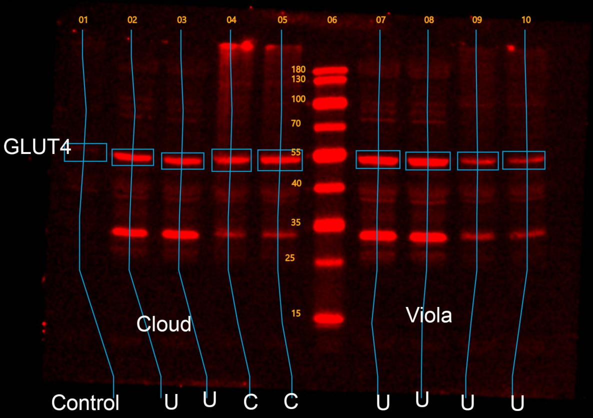
01
02
03
04
05
06
07
08
09
10
180
130
100
70
55
40
35
25
15
GLUT4
Cloud
Viola
Control
U
U
C
C
U
U
U
U

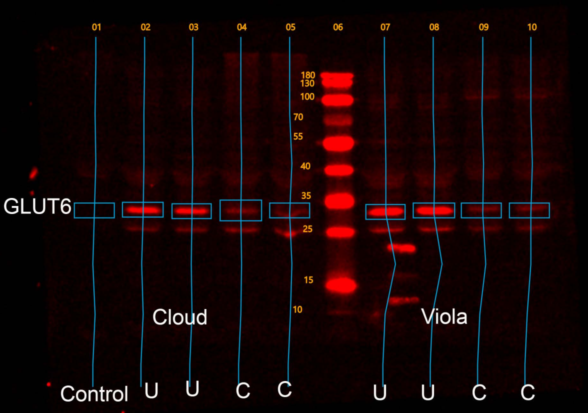
01
02
03
04
05
06
07
08
09
10
180
130
100
70
55
40
35
25
15
10
GLUT6
Cloud
Viola
Control
U
U
C
C
U
U
C
C

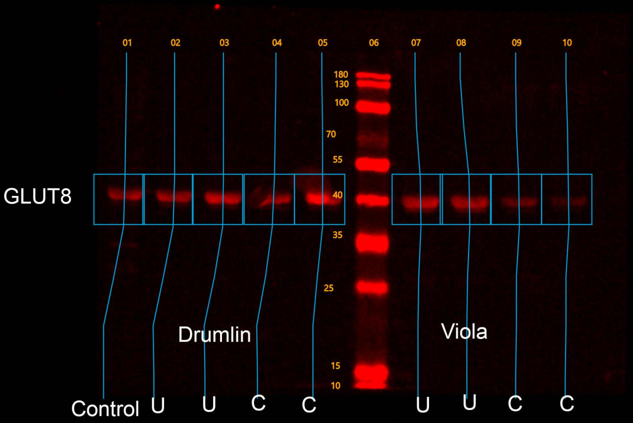

01
02
03
04
05
06
07
08
09
10
180
130
100
70
55
GLUT8
40
35
25
Drumlin
Viola
15
10
Control
U
U
C
C
U
U
C
C

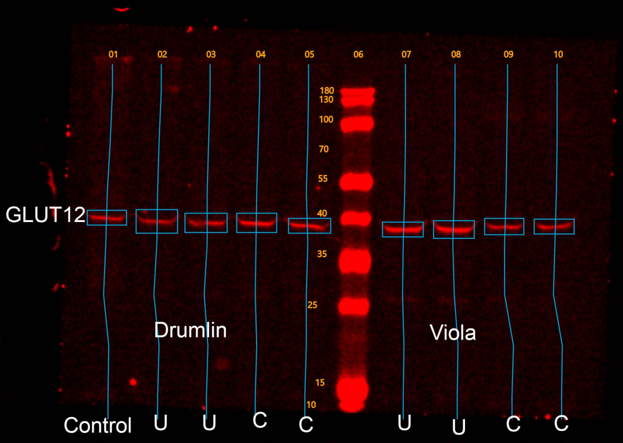

01
02
03
04
05
06
07
08
09
10
180
130
100
70
55
40
35
25
15
10
GLUT12
Drumlin
Viola
Control
U
U
C
C
U
U
C
C

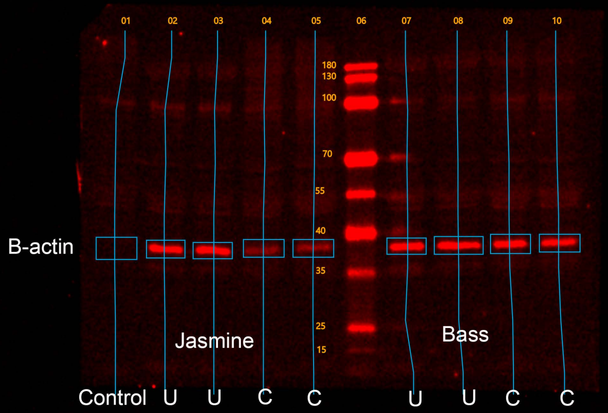
01
02
03
04
05
06
07
08
09
10
180
130
100
70
55
40
35
25
15
B-actin
Jasmine
Bass
Control
U
U
C
C
U
U
C
C

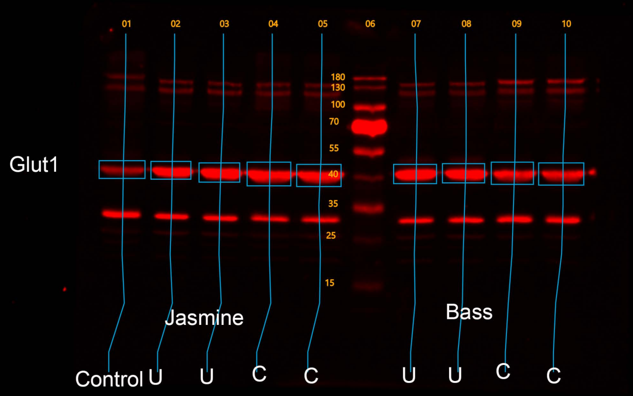
01
02
03
04
05
06
07
08
09
10
180
130
100
70
55
40
35
25
15
Glut1
Jasmine
Bass
Control
U
U
C
C
U
U
C
C

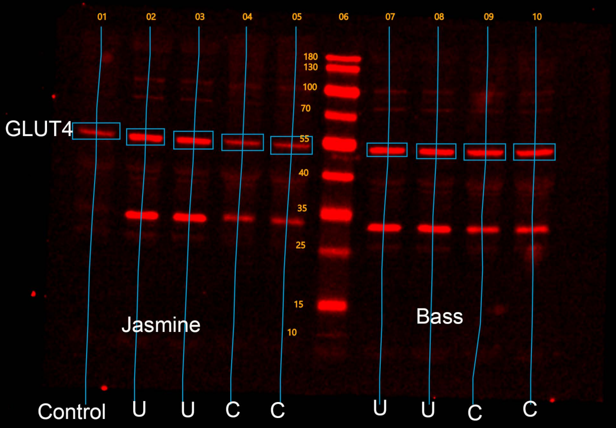
01
02
03
04
05
06
07
08
09
10
180
130
100
70
55
40
35
25
15
10
GLUT4
Jasmine
Bass
Control
U
U
C
C
U
U
C
C

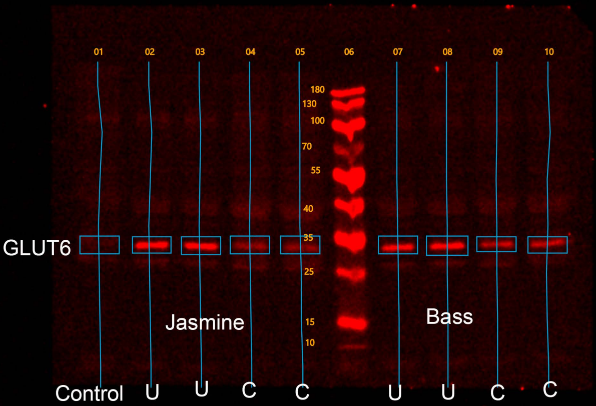

01
02
03
04
05
06
07
08
09
10
180
130
100
70
55
40
35
25
15
10
GLUT6
Jasmine
Bass
Control
U
U
C
C
U
U
C
C

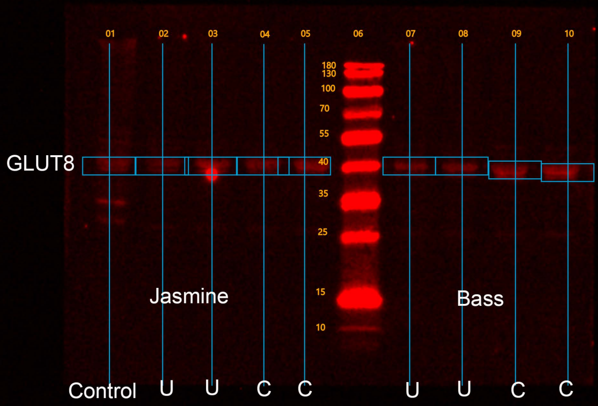

01
02
03
04
05
06
07
08
09
10
180
130
100
70
55
40
35
25
15
10
GLUT8
Jasmine
Bass
Control
U
U
C
C
U
U
C
C

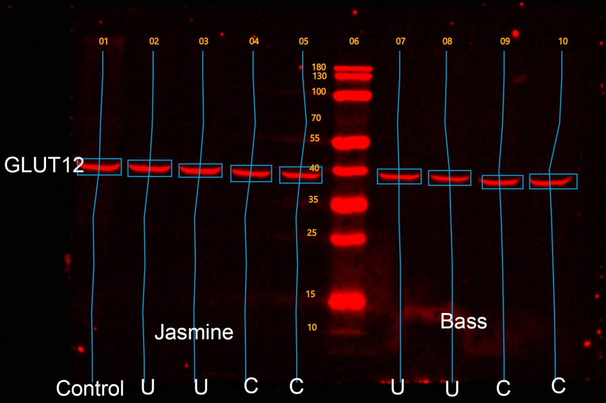

01
02
03
04
05
06
07
08
09
10
180
130
100
70
55
40
35
25
15
10
GLUT12
Jasmine
Bass
Control
U
U
C
C
U
U
C
C

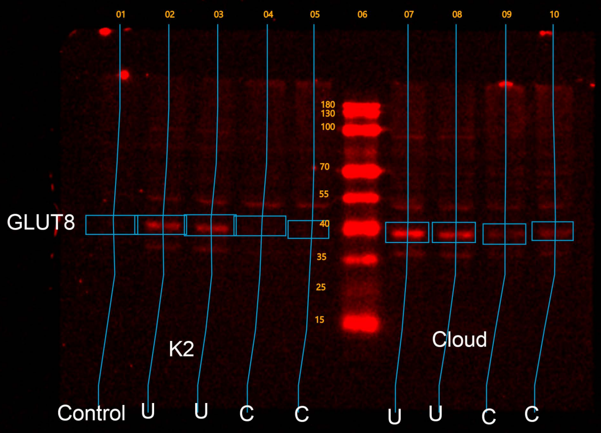
01
02
03
04
05
06
07
08
09
10
180
130
100
70
55
40
35
25
15
GLUT8
K2
Cloud
Control
U
U
C
C
U
U
C
C

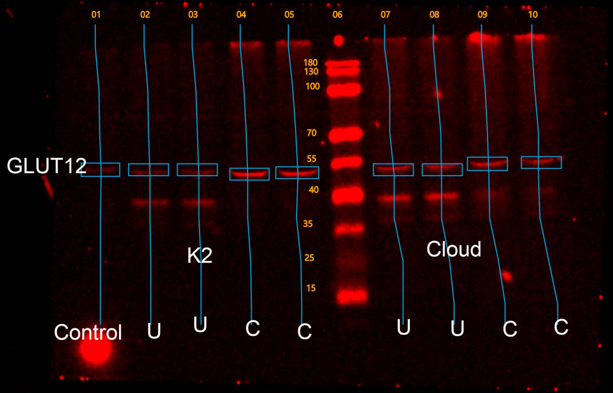
01
02
03
04
05
06
07
08
09
10
180
130
100
70
55
40
35
25
15
GLUT12
K2
Cloud
Control
U
U
C
C
U
U
C
C

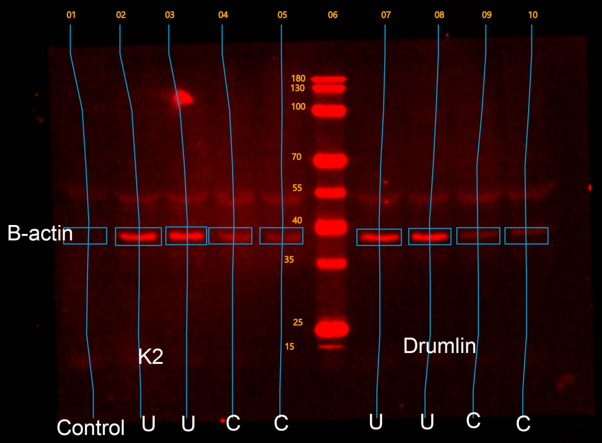
01
02
03
04
05
06
07
08
09
10
180
130
100
70
55
40
35
25
15
B-actin
K2
Drumlin
Control
U
U
C
C
U
U
C
C

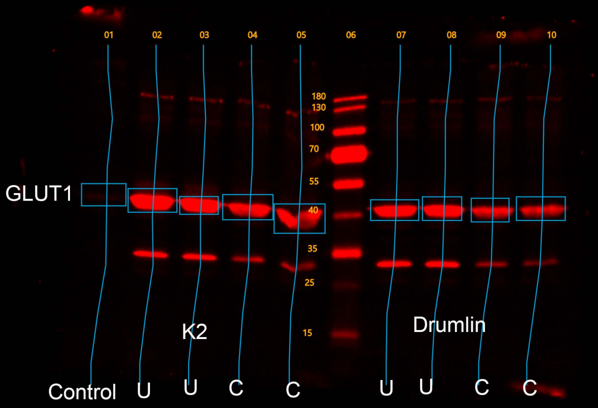

01
02
03
04
05
06
07
08
09
10
GLUT1
180
130
100
70
55
40
35
25
15
K2
Drumlin
Control
U
U
C
C
U
U
C
C

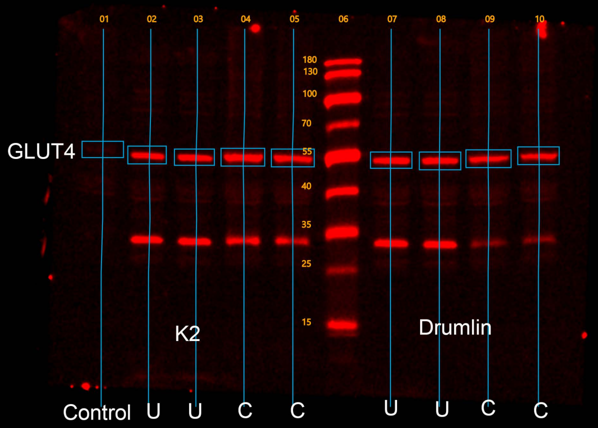

GLUT4
01
02
03
04
05
06
07
08
09
10
180
130
100
70
55
40
35
25
15
K2
Drumlin
Control
U
U
C
C
U
U
C
C

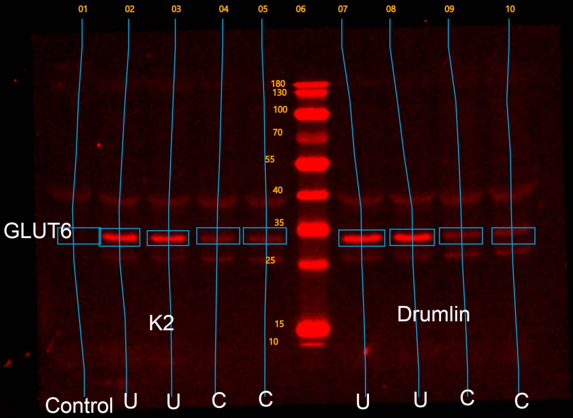
01
02
03
04
05
06
07
08
09
10
180
130
100
70
55
40
35
25
15
10
GLUT6
K2
Drumlin
Control
U
U
C
C
U
U
C
C

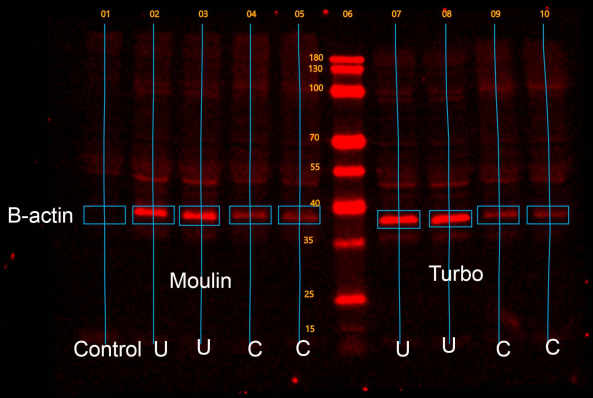
01
02
03
04
05
06
07
08
09
10
180
130
100
70
55
40
35
25
15
B-actin
Moulin
Turbo
Control
U
U
C
C
U
U
C
C

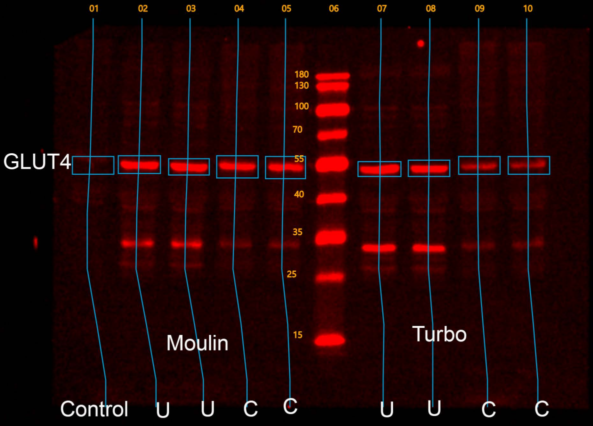

01
02
03
04
05
06
07
08
09
10
GLUT4
180
130
100
70
55
40
35
25
15
Moulin
Turbo
Control
U
U
C
C
U
U
C
C

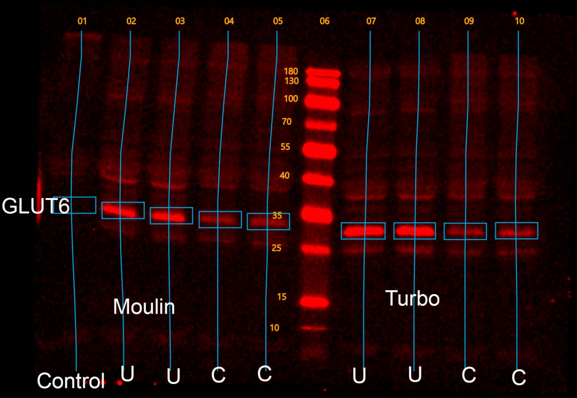

01
02
03
04
05
06
07
08
09
10
180
130
100
70
55
40
35
25
15
10
GLUT6
Moulin
Turbo
Control
U
U
C
C
U
U
C
C

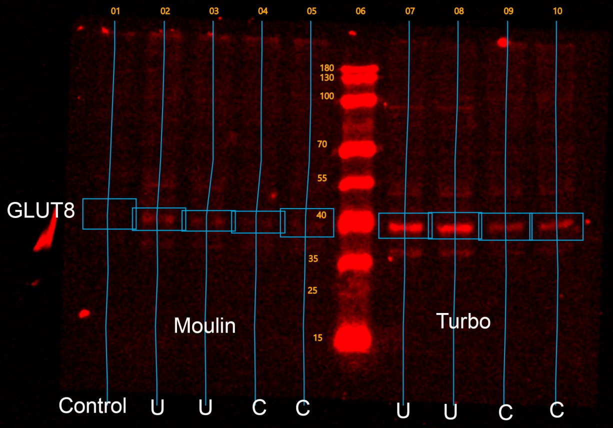
01
02
03
04
05
06
07
08
09
10
180
130
100
70
55
40
35
25
15
GLUT8
Moulin
Turbo
Control
U
U
C
C
U
U
C
C

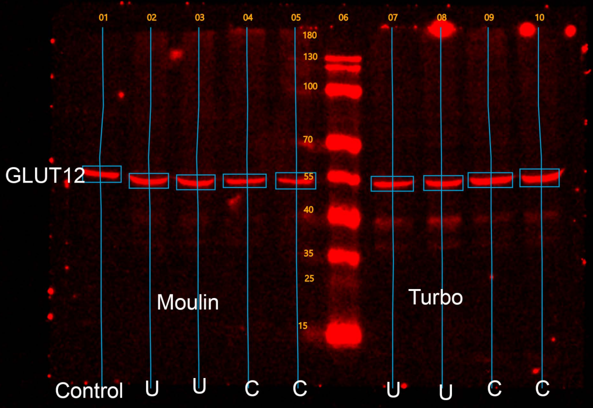
01
02
03
04
05
06
07
08
09
10
180
130
100
70
55
40
35
25
15
GLUT12
Moulin
Turbo
Control
U
U
C
C
U
U
C
C

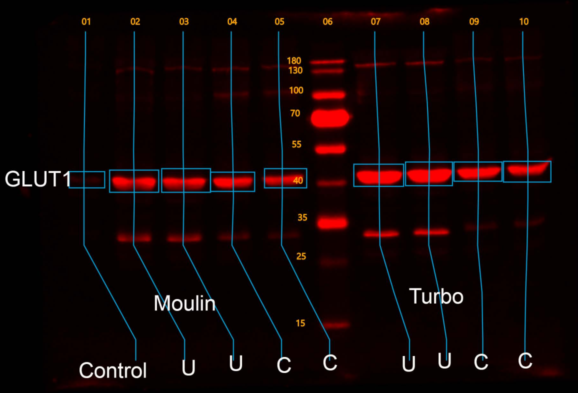
01
02
03
04
05
06
07
08
09
10
180
130
100
70
55
40
35
25
15
GLUT1
Moulin
Turbo
Control
U
U
C
C
U
U
C
C
