# Supplementary material for: Conditioning-induced expression of novel glucose transporters in canine skeletal muscle homogenate
Source: PLoS One. 2023 May 3;18(5):e0285424. doi: 10.1371/journal.pone.0285424 (PMC10155965; doi:10.1371/journal.pone.0285424)
Supplement: S1 Data — (PDF) [file pone.0285424.s002.pdf]

				Raw signal intensity										
Dog	Sex	Date	Beta actin		Glut4		Glut1		Glut6		GLUT8		GLUT12	
Arabica	F	August	0.614	0.531	0.295	0.207	2.15	2.99	0.578	0.478	0.031	0.037	0.388	0.367
Polaris	MN	August	0.382	0.371	0.137	0.196	1.13	0.923	0.326	0.366	0.063	0.074	0.409	0.393
Beans	M	August	0.169	0.185	0.166	0.156	3.67	3.68	0.163	0.163	0.039	0.033	0.295	0.283
Grinder	MN	August	0.303	0.361	0.408	0.332	4.57	4.38	0.248	0.254	0.041	0.043	0.12	0.119
Jasmine	F	August	0.298	0.329	0.369	0.303	4.09	5.09	0.198	0.195	0.095		0.553	0.559
Bass	M	August	0.318	0.392	0.382	0.322	5.32	4.81	0.15	0.182	0.111	0.12	0.4	0.326
Moulin	M	August	0.244	0.257	0.56	0.647	2.99	2.68	0.198	0.192	0.045	0.02	0.266	0.203
Turbo	M	August	0.414	0.416	0.694	0.516	6.13	5.92	0.454	0.447	0.131	0.137	0.197	0.217
K2	MN	August	0.283	0.263	0.352	0.435	5.09	4.2	0.236	0.212	0.85	0.73	0.031	0.029
Drumlin	M	August	0.282	0.287	0.472	0.449	2.82	2.64	0.296	0.298	0.314	0.399	0.09	0.095
Cloud	M	August	0.454	0.432	0.358	0.302	3.19	3.94	0.26	0.236	0.143	0.113	0.058	0.054
Viola	F	August	0.597	0.639	0.599	0.605	3.61	3.23	0.439	0.499	0.42	0.391	0.164	0.171
Arabica	F	March	0.379	0.374	0.178	0.229	2.47	5.19	0.326	0.289	0.047	0.075	0.363	0.436
Polaris	MN	March	0.086	0.081	0.11	0.078	1.18	1.52	0.082	0.059	0.143	0.143	0.371	
Beans	M	March	0.035	0.038	0.275	0.252	1.41	1.4	0.045	0.032	0.096	0.094	0.305	0.203
Grinder	MN	March	0.15	0.134	0.381	0.442	3.96	4.69	0.112	0.077	0.061	0.081	0.101	0.134
Jasmine	F	March	0.105	0.114	0.121	0.12	4.92	4.53	0.074	0.07	0.142	0.185	0.463	0.425
Bass	M	March	0.281	0.234	0.275	0.29	3.44	3.44	0.098	0.117	0.198	0.232	0.348	0.4
Moulin	M	March	0.107	0.097	0.412	0.39	2.55	1.93	0.086	0.088	0.012	0.018	0.168	0.129
Turbo	M	March	0.087	0.068	0.174	0.157	3.26	2.33	0.075	0.093	0.045	0.061	0.409	0.379
K2	MN	March	0.087	0.094	0.598	0.454	2.42	2.81	0.053	0.069	0.004	0.001	0.106	0.099
Drumlin	M	March	0.05	0.053	0.399	0.345	1.55	1.56	0.064	0.063	0.299		0.127	0.112
Cloud	M	March	0.099	0.072	0.267	0.371	2.82	2.3	0.081	0.069	0.036	0.038	0.064	0.054
